# Real‐Time Identification of Lymph Vessels Using Indocyanine Green in a Patient With Chylothorax Associated With Lymphangioleiomyomatosis

**DOI:** 10.1111/ases.70067

**Published:** 2025-04-20

**Authors:** Shinichi Sakamoto, Hiroaki Toba, Ayaka Baba, Emi Takehara, Keisuke Fujimoto, Taihei Takeuchi, Hiroyuki Sumitomo, Naoki Miyamoto, Atsushi Morishita, Naoya Kawakita, Hiromitsu Takizawa

**Affiliations:** ^1^ Department of Thoracic and Endocrine Surgery and Oncology, Institute of Biomedical Sciences Tokushima University Graduate School Tokushima Japan

**Keywords:** chylothorax, indocyanine green, lymphangioleiomyomatosis

## Abstract

**Introduction:**

Lymphangioleiomyomatosis (LAM) is often complicated by chylothorax and may require surgical intervention; however, the treatment is complicated because of difficulties in identifying the location of the fistula intraoperatively. This is the first report to identify the site of a chyle fistula associated with LAM in real time during surgery by using indocyanine green (ICG) lymphangiography.

**Materials and Surgical Technique:**

A 56‐year‐old woman received a diagnosis of a treatment‐resistant left chylothorax associated with LAM. To identify the chyle fistula during surgery, 1 mL of ICG (2.5 mg) was injected into both inguinal lymph nodes under ultrasound guidance after anesthesia, with 1 mL per side for a total of 5 mg of ICG. We performed video‐assisted thoracic surgery and observed near‐infrared light acquisition and overlay technology using Stryker. Approximately 1 h after administration, fluorescence was observed in the anterior mediastinal lymph nodes, and a chyle fistula was observed around them. Although we attempted ligation of the lymph trunk, the surgical procedure damaged well‐developed lymph vessels. The damaged area and anterior mediastinal lymph nodes, including the surrounding lymph vessels, were incinerated using soft coagulation and covered with polyglycolic acid sheets and fibrin glue. Consequently, the amount of chylous effusion decreased.

**Discussion:**

The use of ICG allowed visualization of the lymphatic pathway and location of the chyle fistula in real time during surgery, enabling precise local treatment to reduce chyle effusion.

## 1 Introduction

Lymphangioleiomyomatosis (LAM) is a rare disease affecting women of childbearing age. LAM primarily manifests as diffuse pulmonary cysts in the lungs, with the proliferation of smooth muscle cell‐like LAM cells in lymphatic tissues and kidneys. The main symptoms of LAM are respiratory complications, including dyspnea and recurrent pneumothorax; other symptoms include renal angiomyolipoma and chylothorax [1, 2]. Chylothorax occurs in approximately 10% of cases [1], and conservative treatment is generally attempted first; if ineffective, more invasive treatments are considered. In cases of nontraumatic chylothorax, identifying the chyle fistula site during surgery is difficult; blind surgery without knowledge of the anatomy of lymph vessels (i.e., the thoracic duct) underlying the disease is dangerous and should be avoided [3]. Herein, we report a case in which lymph vessels and the chyle fistula were identified during surgery using ICG for treatment‐resistant chylothorax associated with LAM.

## 2 Materials and Surgical Technique

**FIGURE 1:**
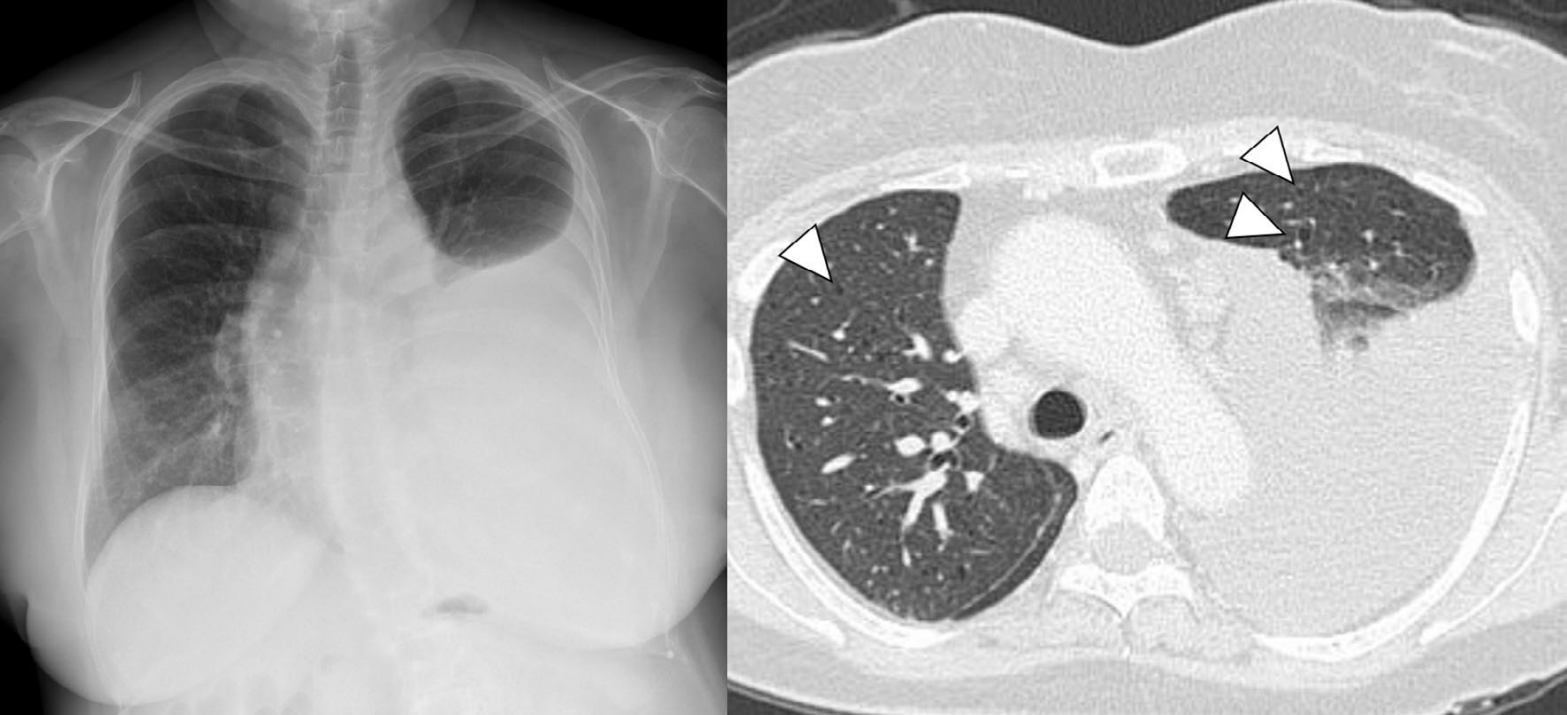
Chest radiograph revealed a left pleural effusion, and computed tomography revealed multiple thin‐walled cysts in both lung fields (white arrowheads).

A 56‐year‐old woman was referred to our hospital for evaluation of left pleural effusion and dyspnea. She had a history of transcatheter arterial embolization for left renal angiomyolipoma. Chest radiography and computed tomography revealed a left pleural effusion with multiple thin‐walled cysts in both lung fields (Figure 1). Thoracentesis yielded a milky fluid; the condition was confirmed to be chylothorax based on a triglyceride level of 2998 mg/dL and absence of dysplastic cells in the fluid. Based on these clinical features, we suspected LAM.

**FIGURE 2:**
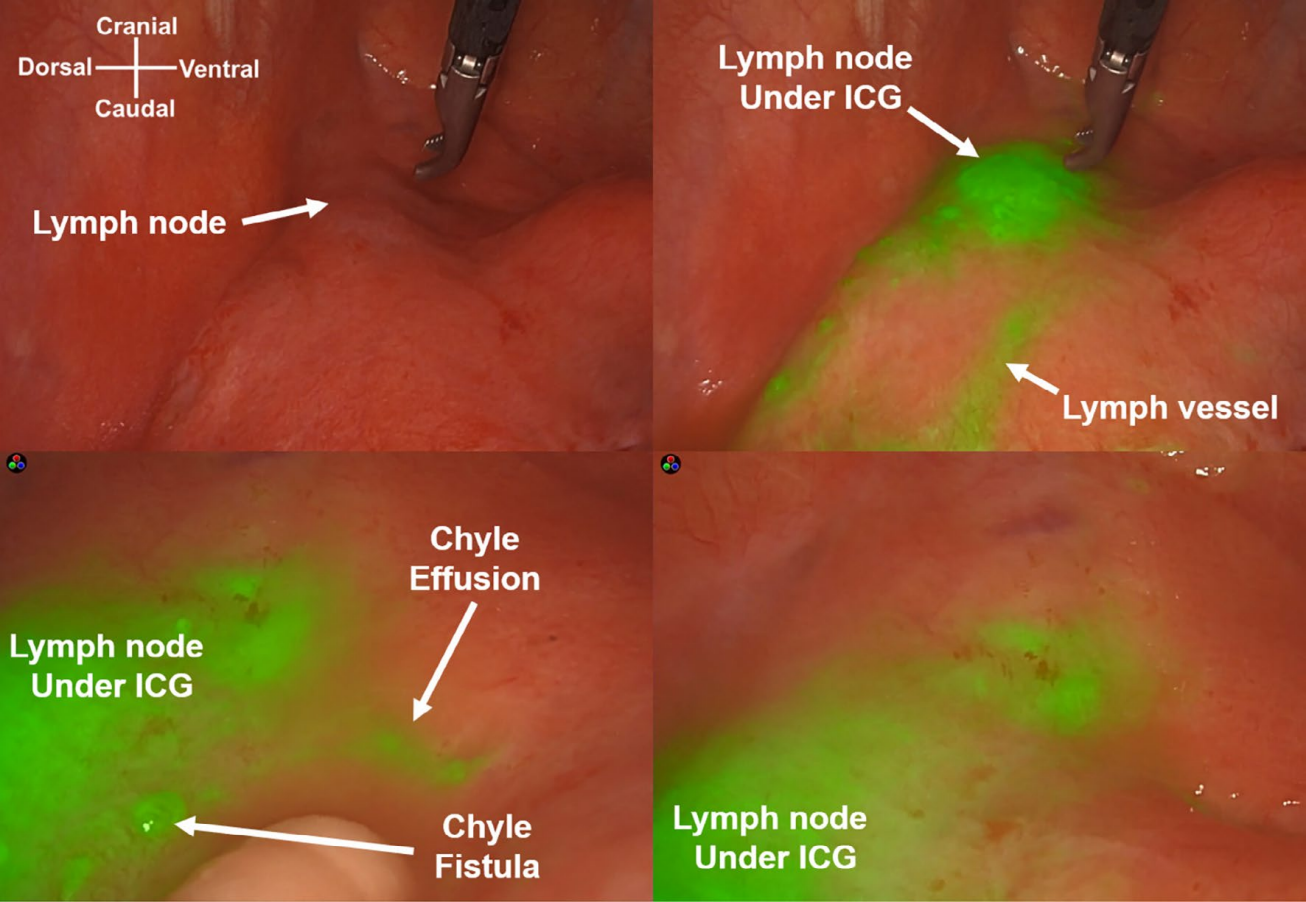
Intraoperative indocyanine green (ICG) imaging of lymph node and lymph vessels (superior view). The anterior mediastinal lymph nodes and lymph vessels developed around them were fluorescent, and a chyle fistula was observed around them (inferior view).

**FIGURE 3:**
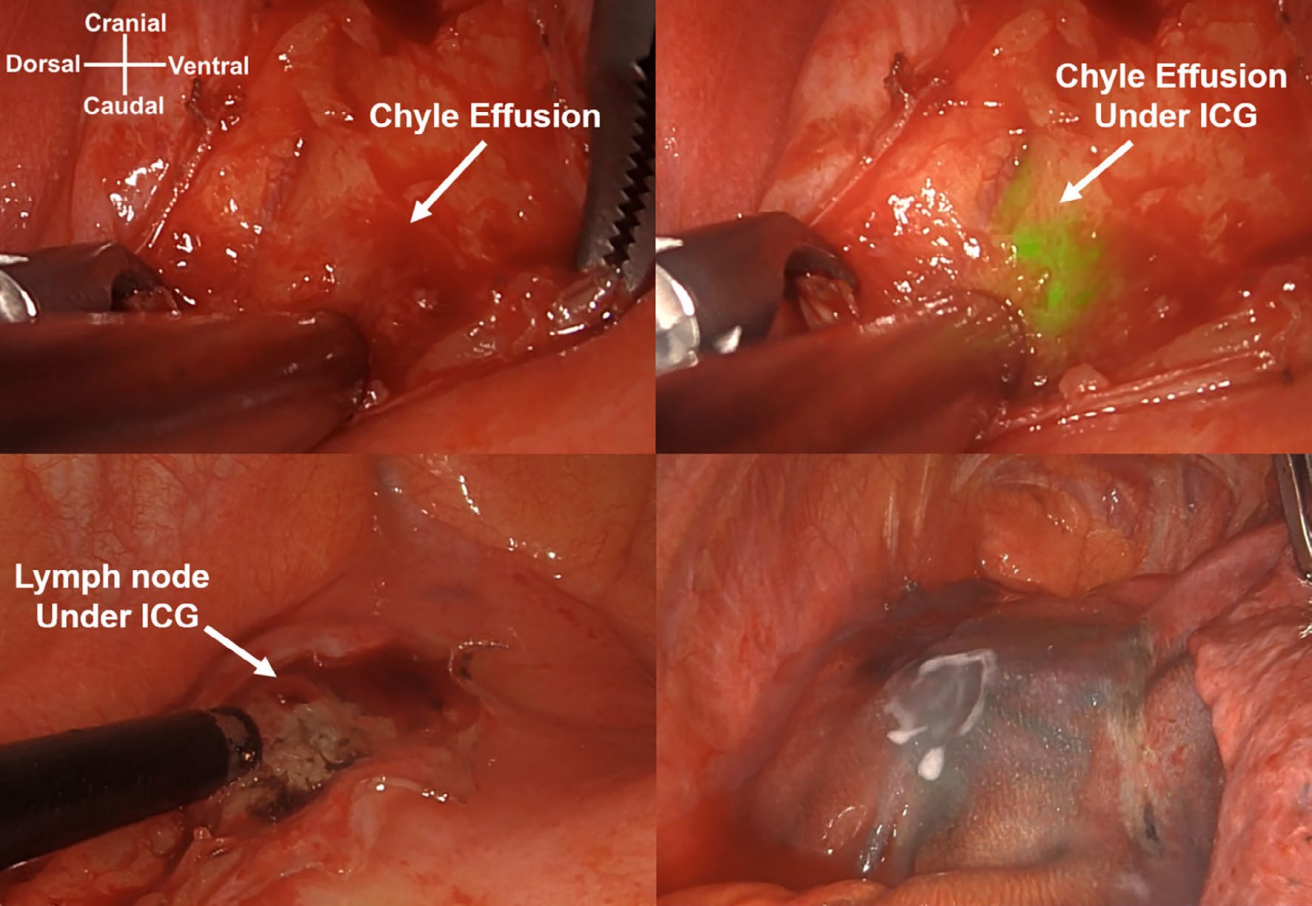
Intraoperative surgical manipulation of the chyle fistula. We attempted to ligate the lymph trunk; however, the chyle effusion leaked from the developed lymphatic tissue (superior view). The chyle fistula was treated with soft coagulation, and the coagulated area was reinforced with a polyglycolic acid sheet and fibrin glue to prevent recurrence (inferior view).

As drainage and low‐fat diet treatment was ineffective, video‐assisted thoracic surgery was performed on the left side. To visualize the chyle fistula, ICG (Diagnogreen; Daiichi Sankyo, Tokyo, Japan) was injected in the inguinal nodes bilaterally (each side 1 mL, total 5 mg) under sonographic guidance after anesthesia. ICG is available as a 25‐mg powder and reconstituted in 10 mL of water [4]. We used a 30° camera equipped with near‐infrared light acquisition and overlay technology (1688 AIM 4 K platform; Stryker, Kalamazoo, MI, USA). Approximately, 1600 mL of chyle pleural effusion was observed in the thoracic cavity. The anterior‐mediastinal lymph nodes located ventral to the phrenic nerve and lateral to the ascending aorta (#6 Ln) and developed lymph vessels fluoresced, and chylous effusion leaked from the edge of the lymph node approximately 1 h after the ICG injection (Figure 2). We attempted to ligate the lymph trunk; however, the tiny proliferating lymph vessels were disrupted by the surgical procedure. Therefore, the damaged area and network of the lymph vessels were incinerated using soft coagulation and covered with polyglycolic acid sheets and fibrin glue (Figure 3; Video S1). The left lower lobe pleura was torn because of re‐expansion, and wedge resection was performed for biopsy and repair. The operative time was 194 min, and the amount of blood loss was 5 mL. A low‐fat diet was initiated on postoperative Day 2. Although the pleural effusion volume decreased to 50 mL/day, pleurodesis was performed with 5KE of OK‐432 (Picibanil, Chugai: Pharmaceutical, Tokyo, Japan) 3 days after surgery because the fluid triglyceride level was high (331 mg/dL). The pleural effusion decreased to 10 mL/day; the chest tube was removed on Day 12, and the patient was discharged 14 days after surgery.

**FIGURE 4:**
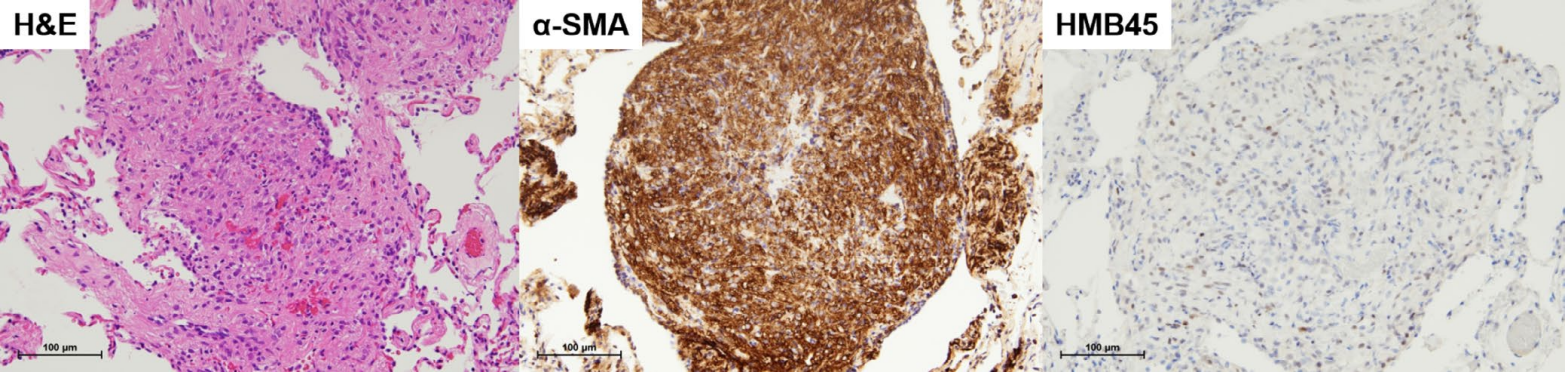
Histological findings of the lymphangioleiomyomatosis (LAM) lesions in the lung. hematoxylin and eosin (H&E) staining (scale bars, 100 μm) revealed proliferation of spindle‐shaped LAM cells in multiple nodules in the lung tissue. Immunohistochemically, short spindle‐shaped cells are positive for α‐smooth muscle actin (α‐SMA) and human melanin black (HMB45) (scale bars, 100 μm).

Immunohistochemical examination revealed LAM with α‐smooth muscle actin‐positive and human melanin black 45‐positive short spindle‐shaped cells (Figure 4). In this case, the causative gene for tuberous sclerosis complex had not been identified; thus, the patient received a diagnosis of sporadic LAM [5]. Chylothorax did not recur at the 6‐month follow‐up visit.

## 3 Discussion

Here, we report the first case of LAM complicated by chylothorax, wherein lymph vessels were identified using ICG in real time during surgery. ICG helped us identify chyle fistulae and perform critical surgical procedures.

Persistent chylothorax requires aggressive treatment, because it can be fatal due to respiratory complications, immunosuppression, and nutritional wastage, with a mortality rate of 25%–50% [6]. The thoracic duct has numerous variations [6], making the treatment of nontraumatic chylothorax more challenging than that of traumatic or iatrogenic chylothorax treatment [7]. CT lymphangiography in patients with LAM revealed various lymphatic abnormalities; the leakage site was identified in only 33% of patients with chylothorax [8]. Other methods, including preoperative lymphangiography and lymphoscintigraphy, are used to identify the chyle fistula; however, it is difficult to directly transfer anatomical information to the intraoperative situation [9], and the location of the chyle fistula varies among cases. No previous reports have described accurate real‐time identification of the leakage site during surgery; therefore, our method of using ICG is a breakthrough. If the chyle fistula site is unknown, the thoracic duct is ligated above the right diaphragm. Nonetheless, in our case, as the chylothorax had accumulated on the left side of the thoracic cavity, we decided to approach it from that side. Although the chyle fistula site can be identified by consuming olive oil or dairy products before surgery [10, 11], the contrast is often low, and identification can be difficult [12]. Hence, we injected ICG into both inguinal lymph nodes to visualize them during surgery. In this case, although a chyle fistula was predicted in the posterior mediastinum above the diaphragm, we found it around the enlarged anterior mediastinal lymph nodes, and no lymph vessels were visualized in the posterior mediastinum. ICG enabled the intraoperative visualization of lymph vessels with high reproducibility [4], thus preventing blind and careless surgical procedures. Although we could not ligate the lymph trunk, local surgical procedures were performed to reduce the volume of chylous effusion, allowing us to proceed to the next stage of treatment. As pleurodesis is ineffective for massive pleural effusion [13], reducing the volume of chylous effusion through surgical treatment has led to favorable results. Nontraumatic chylothorax is more difficult to treat and requires multidisciplinary therapy [14]. ICG allows the accurate identification of chyle fistulae, enabling appropriate surgical treatment. Lymphangiography using ICG is a useful surgical treatment for chylothorax.

## Author Contributions

Writing – original draft: Shinichi Sakamoto. Data curation: Ayaka Baba, Emi Takehara, and Keisuke Fujimoto. Investigation: Shinichi Sakamoto and Atsushi Morishita. Supervision: Naoya Kawakita and Hiromitsu Takizawa. Visualization: Taihei Takeuchi, Naoki Miyamoto, and Hiroyuki Sumitomo. Writing – review and editing: Shinichi Sakamoto and Hiroaki Toba. All of the authors have read and approved the final manuscript.

## Ethics Statement

This study was approved by the Research Ethics Committee of our hospital. The patient's identity was protected.

## Consent

The patient provided written informed consent for publication of this case report.

## Conflicts of Interest

The authors declare no conflicts of interest.

## Supporting information


**Video S1.** Video‐assisted thoracic surgery performed using a 30° camera equipped with near‐infrared light acquisition and overlay technology (1688 AIM 4 K platform; Stryker, Kalamazoo, MI, USA). Fluorescence was observed in the anterior mediastinal lymph nodes (#6 Ln) after 1 h, with a chyle fistula around them. The chyle fistula was disrupted owing to surgical damage to the lymph vessels, which were treated with soft coagulation, polyglycolic acid sheets, and fibrin glue.

## Data Availability

The data that support the findings of this study are available on request from the corresponding author. The data are not publicly available due to privacy or ethical restrictions.
